# Cryptococcus neoformans in Chronic Lymphocytic Leukemia (CLL) Treated With Ibrutinib: A Combo Gone Wrong!

**DOI:** 10.7759/cureus.81113

**Published:** 2025-03-24

**Authors:** Yogesh Yadav, Felipe Centellas Montano, Elizabeth Plocharczyk, Douglas MacQueen, SushilKumar Gupta

**Affiliations:** 1 Internal Medicine, Cayuga Medical Center, Ithaca, USA; 2 Pathology, Cayuga Medical Center, Ithaca, USA; 3 Infectious Disease, Cayuga Medical Center, Ithaca, USA; 4 Pulmonary and Critical Care, Cayuga Medical Center, Ithaca, USA

**Keywords:** b cell immunotherapy, bruton’s tyrosine kinase, chronic lymphocytic leukemia, cryptococcus neoformans, ibrutinib

## Abstract

A 71-year-old female, with hypertension, depression, and asthma, was diagnosed with chronic lymphocytic leukemia (CLL) (elevated beta-2 microglobulin and deletion of 13 q chromosome) and treated with ibrutinib. She had general weakness, dyspnea, fatigue, shortness of breath, upper abdominal fullness, and discomfort from splenomegaly. In the ED, her oxygen saturation (SpO_2_) was 83% on room air, afebrile, and stable. Nasal oxygen at 3 L improved her hypoxemia and symptoms. An exam showed bilateral crackles, systolic murmur 2/6, non-tender abdomen with splenomegaly, and no lower extremities edema. A CT scan showed patchy ground glass opacities, interlobular septal thickening, and bilateral pleural effusion. Diuretics were given, but the patient's condition worsened, requiring ICU and bi‐level positive airway pressure (BiPAP). Staphylococcus hominis was found in blood cultures, and vancomycin was started. Respiratory distress necessitated mechanical ventilation and intubation. Imaging suggested acute respiratory distress syndrome (ARDS), atypical pneumonia, or heart failure with preserved ejection fraction. Transthoracic echocardiogram showed normal ejection fraction with severely increased pulmonary artery pressure, and transesophageal echocardiogram showed no vegetation. Blood cultures monitored the Gram-positive bacteremia, with BioFire isolating Candida zeylanoides initially. Subsequent cultures over a week period were positive for C. zeylanoides and then Cryptococcus neoformans. Chest CT revealed a large mass of lymph nodes in the mediastinal area, which was thought to be causing pulmonary hypertension by compressing the pulmonary artery. After a multidisciplinary discussion, ibrutinib was withheld, and drug-induced pneumonitis was suspected. Fungemia was found in the immunocompromised patient, so the infectious disease team prescribed voriconazole for 13 days and then changed to amphotericin and fluconazole due to a lack of improvement. Eye examination showed no signs of endophthalmitis. Lumbar puncture showed no central nervous system (CNS) fungal infection. The patient's respiratory status worsened, so a bronchoscopy with bronchoalveolar lavage (BAL) of the right middle lobe and chest tube placement for pleural effusion was done. Microbiological examination of BAL and pleural effusion revealed C. neoformans and Klebsiella, confirming disseminated C. neoformans. Despite a tough 32-day hospital stay, the patient was discharged in stable condition. Physical therapy and nutrition optimization were used to enhance her health. This case report highlights the rare but serious complication of cryptococcal disease in patients using ibrutinib for blood cancers. Early comprehensive diagnosis and multi-disciplinary involvement saved our patient's life.

## Introduction

Chronic lymphocytic leukemia (CLL) is a type of lymphoid neoplasm manifesting in blood characterized by monoclonal functionally incompetent lymphocytes, whereas small lymphocytic lymphoma (SLL) primarily involves lymph nodes [1]. Patients are usually diagnosed approximately at the age of 70 years; however, younger individuals in the age group of 30-39 years may also be involved ​​​​​[1]. The recommended treatment is ibrutinib for CLL in these groups of patients [2]. Ibrutinib is an antineoplastic agent that inhibits Bruton’s tyrosine kinase (BTK), which is a signaling molecule of the B-cell antigen receptors that results in activation pathways for B cells [3]. Nonclinical studies have shown in vivo that survival and malignant proliferation are inhibited by ibrutinib, and in vitro data suggest that it inhibits not only B-cell migration but also substrate adhesion [4]. Practitioners are aware of complications such as infections (upper respiratory infections, progressive multifocal leukoencephalopathy, and Pneumocystis jirovecii pneumonia), hemorrhage, cytopenias, atrial fibrillation, and tumor lysis syndrome. In post-marketing surveys, patients were also found to have hepatobiliary disorders, interstitial lung disease, Stevens-Johnson syndrome, and onycholysis. We present here a rare association of fungemia (Cryptococcus neoformans) in the patient treated with ibrutinib for CLL. The practitioners have to be vigilant of this life-threatening association, which will also help in early diagnosis and prompt management. This association is consistent with other case reports on this finding [5].

## Case presentation

A 71-year-old female, with a past medical history of hypertension, depression, and asthma, recently diagnosed with chronic lymphocytic leukemia, presented with a weeklong history of generalized weakness and a three-day history of progressive shortness of breath. Two months prior to admission to the hospital, the patient was noted to have bilateral cervical adenopathy, a biopsy of one of the lymph nodes revealed a CD5-positive B-cell malignancy consistent with small lymphocytic lymphoma/chronic lymphocytic leukemia. Thepatient was referred to hematology/oncology, and CBC revealed a white count of 164,000 x 10^9^/L with predominantly lymphocytes, severe anemia with a hemoglobin of 6 g/dL, and moderate thrombocytopenia with a platelet count of 75,000/µL. Lactate dehydrogenase (LDH) was within normal limits. Beta-2 microglobulin was elevated at 11 mcg/mL. Fluorescent in situ hybridization (FISH) studies revealed deletion 13q chromosome, which is a good prognostic factor, and no evidence of 17 P deletion, TP 53 mutation, or 11;14 translocation, which are poor prognostic factors [6,7]. The patient was given a transfusion of ibrutinib at a reduced dose given her concomitant calcium channel blocker diltiazem as both are metabolized by CYP3A4 liver enzymes and diltiazem inhibits CYP3A4 enzyme and can increase the concentration of ibrutinib. The patient was also found to have elevated uric acid most likely due to tumor lysis syndrome in the setting of recent treatment with ibrutinib and was started on febuxostat concomitantly. The patient appeared to be tolerating therapy and responding with a decrease in her lymphocyte count and tumor burden.

One week before admission, she felt more fatigued, and this progressed to shortness of breath, for which she went to the emergency room and was found to be hypoxic. She reported having upper abdominal fullness and discomfort due to splenomegaly from the lymphoma. In the ED, her O_2_ saturation was initially 83% on RA, afebrile, and hemodynamically stable. The patient was placed on nasal oxygen at 3 L with improvement in her hypoxemia and symptoms.

Physical exam

On admission, the patient presented with a temperature of 98.1 °F, pulse rate of 63/min, blood pressure (BP) of 124/56 mmHg, relative risk (RR) of 26/min, and oxygen saturation (SpO_2_) of 97% with 3 L of supplemental oxygen. Remarkable on the physical exam, she was a pleasant female, with the presence of a left-sided 1 x 1-inch submandibular lymph node enlargement, no evidence of JVD, bilateral crackles worst at the pulmonary bases, decreased air movement over the right middle and lower lung zones, tachypnea, no wheezing, heart auscultation with regular rhythm, presence of a systolic 2/6 murmur, abdomen with a non-tender splenomegaly four finger breadth below the rib cage, no lower extremities edema noted, and no focal neurological deficit.

Course of treatment

**Figure 1:**
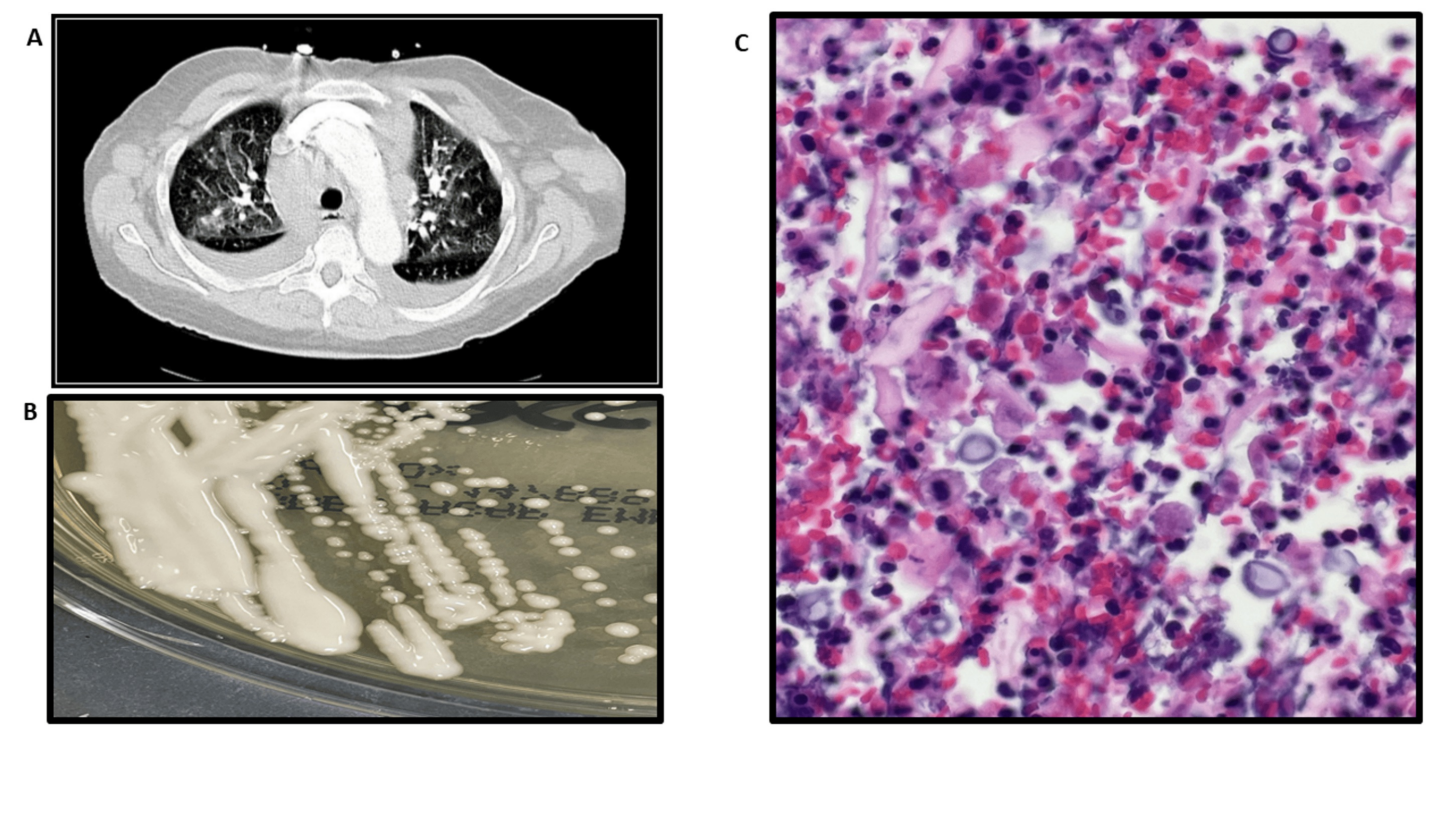
(A) CT chest showing patchy ground glass opacification throughout the lungs bilaterally. (B) Mucoid white colonies growing on sabouraud agar. (C) Low-power H&E stain showing mucoid capsules of varying shapes and sizes.

A CT scan of the chest showed diffuse patchy ground glass opacities, ground glass opacification with diffuse interlobular septal thickening, and bilateral pleural effusion (Figure 1A). There was also a concern for volume overload, so she was administered diuretics. The patient had transient improvement but rapidly deteriorated, prompting the transfer to the intensive care unit due to the need for rescue bi‐level positive airway pressure (BiPAP).

The initial blood cultures resulted in a positive for Staphylococcus hominis, and she was started on vancomycin. However, her respiratory distress progressively deteriorated, requiring mechanical ventilation and endotracheal intubation due to the persistence of high oxygen requirements, and new imaging was concerning for acute respiratory distress syndrome (ARDS) vs atypical pneumonia vs heart failure with preserved ejection fraction.

New sets of blood cultures were obtained to monitor the Gram-positive bacteremia; with the BioFire technique, they were isolated initially as Candida zeylanoides, and subsequent cultures between a week period continued to be positive for this and for C. neoformans (Figure 1B, Figure 1C).

An initial transthoracic echocardiogram reported a patent foramen ovale (PFO) and severe pulmonary hypertension; this was confirmed with a transesophageal echocardiogram that was obtained to rule out infective endocarditis, where it did not show vegetation but confirmed the previous findings. On computed tomography imaging of the chest, there was a large burden of lymph nodes in the mediastinal area encompassing the pulmonary artery. This was thought to be a plausible cause of severe pulmonary hypertension due to external compression of the pulmonary artery by the tumor. After a multi-disciplinary discussion, a decision was made to hold ibrutinib, and an initial diagnosis was presumed to be drug-induced pneumonitis. Nonetheless, later, the fungemia was identified in the presence of severe immunosuppression. After consulting with the infectious disease team, the patient was started on voriconazole for 13 days but was changed to amphotericin and fluconazole due to a lack of improvement. On eye examination, there was no concern for endophthalmitis. A lumbar puncture was negative for the central nervous system (CNS)-invasive fungal infection. Due to worsening respiratory status, the patient underwent bronchoscopy with bronchoalveolar lavage (BAL) of the right middle lobe and involved segments and chest tube placement for the management of pleural effusion. Microbiology of BAL and pleural effusion revealed C. neoformans and Klebsiella, now confirming our diagnosis of disseminated C. neoformans. The patient also received seven days of piperacillin/tazobactam in the meantime.

During her illness, the patient showed gradual improvement and was extubated to a high-flow nasal cannula. However, during her recovery phase, she developed maculopapular rash that was thought to be related to amphotericin. A punch biopsy showed perivascular dermatitis with eosinophils. However, given that amphotericin was felt to be the only option, the antifungal agent was continued until her cultures were negative, along with fluconazole as a part of induction therapy. She was discharged on fluconazole 800 mg PO daily for eight weeks, followed by maintenance therapy with fluconazole 400 mg PO daily for one year.

## Discussion

It is well known that C. neoformans infection has been associated with immunocompromised patients; however, there are sparse data about the development of C. neoformans in patients receiving ibrutinib [5]. We report a case that developed after ~6 weeks of treatment from ibrutinib for CLL. The Naranjo algorithm calculator shows a possible adverse reaction with the drug ibrutinib causing cryptococcosis. Cryptococcal infections typically arise due to a flaw in cell-mediated immunity, particularly lymphopenia, although humoral immunity also plays a significant role [8]. X-linked immunodeficient mice, which carry a mutation causing a defective BTK, exhibit a substantial fungal load following experimental exposure to C. neoformans, with the fungus spreading to the brain. This indicates that therapies targeting BTK could potentially increase the risk of cryptococcosis [9]. Our patient had disseminated cryptococcosis, and her primary source was pulmonary cryptococcal infection. The development of the disease might be linked to a decreased uptake of glucuronoxylomannan by macrophages, which is the primary component of the C. neoformans capsule. This reduction could be induced by tyrosine kinase inhibitors. Consequently, this may result in a detrimental interaction between glucuronoxylomannan, a crucial virulence factor of the yeast, and the binding sites on cells that are vital for the host’s immune defense [10]. Although our patient was started on a low dose of ibrutinib as she was on a calcium channel blocker for the reason mentioned above, a study published by Varughese et al. did not show any association between the dose of ibrutinib and the risk of developing serious infections [11]. Lumbar puncture did not show any invasive cryptococcal CNS infection. Immunoglobulin (IgG and IgM) were found to be low. Cryptococcus antigen titers were very high (>1:2560) (Table 1) prior to treatment, which was undetected after seven days of treatment with amphotericin B. Her WBCs were 94 x 10^9^/L, which came back to normal at 4.1 x 10^9^/L after the treatment at the time of discharge. 

**Table 1:** Laboratory parameters. AST: aspartate aminotransferase; ALT: alanine aminotransferase; LDH: lactate dehydrogenase; CSF: cerebrospinal fluid

Hematology	Reference Range	Initial Labs	Peak/Nadir Abnormal Value	At Discharge
White Blood Cell (WBC) (×10⁹/L)	4.0–11.0	94.9	94.9	4.1
Absolute Lymphocyte Count (×10⁹/L)	1.0–4.8	84.8	84.8	1.5
Hemoglobin (g/dL)	13.0–17.0	8.2	6.4	8.0
Platelets (×10⁹/L)	150–450	130	30	95
Biochemistry				
Blood Urea Nitrogen (BUN) (mg/dL)	7–20	37	90	42
Creatinine (mg/dL)	0.6–1.3	1.00	1.65	0.91
Total Bilirubin (mg/dL)	0.1–1.2	0.7	0.7	0.5
AST (U/L)	10–40	8	5	16
ALT (U/L)	7–56	7	4	33
LDH (U/L)	140–280	168	175	175
Inflammatory & Infectious Markers				
C-Reactive Protein (CRP) (mg/L)	<8	74	186	17
Cryptococcal Antigen (Titer)	Negative	>=1:2560	>=1:2560	Negative
CSF Total Cells (Cells/μL)	<5	4	NA	NA
CSF Protein (mg/dL)	15–45	35	NA	NA
CSF Glucose (mg/dL)	50–80	56	NA	NA
CSF Cryptococcal Antigen (Titer)	Negative	Negative	NA	NA
Microbiology				
Blood Cultures	No growth	Candida zeylanoides and Cryptococcus neoformans	Cryptococcus neoformans	No growth
Fungal Stains (BAL)	No growth	Cryptococcus neoformans	Cryptococcus neoformans	NA
Fungal Stains (Pleural Fluid)	No growth	Cryptococcus neoformans	Cryptococcus neoformans	NA

Our patient had a very complicated and rough course during her hospitalization of 32 days and was eventually discharged in clinically and hemodynamically stable condition. She did require a significant amount of physical therapy due to deconditioning. Her nutrition was also optimized to meet her calorie needs and promote strength.

## Conclusions

With this case report, we want clinicians to be aware of this rare life-threatening complication and to keep cryptococcal disease as a potential differential in patients who are treated with ibrutinib for their hematological malignancies. Early involvement of a multi-disciplinary approach, comprehensive workup plan, and prompt diagnosis could save someone’s life like our patient.
